# Supplementary material for: Involvement of Laccase2 in Cuticle Sclerotization of the Whitefly, Bemisia tabaci Middle East–Asia Minor 1
Source: Insects. 2022 May 18;13(5):471. doi: 10.3390/insects13050471 (PMC9144992; doi:10.3390/insects13050471)
Supplement: Supplementary file 1 [file insects-13-00471-s001.zip › insects-1696334-supplementary.pdf]

```
             10         20         30         40         50         60         70         80         90        100        110        120
1      ATGAAGGTGAAAATGTCACGATCAAACGGATTATTTGTGGTGCTCGTTATTGGTGTTTGCTGTGAGTTTGTAAGTTCTCAAGATATCCTCAAGACGTCGACGGCGTCCGCAAAGGACGGG
1        M  K  V  K  M  S  R  S  N  G  L  F  V  V  L  V  I  G  V  C  C  E  F  V  S  S  Q  D  I  L  K  T  S  T  A  S  A  K  D  G
            130        140        150        160        170        180        190        200        210        220        230        240
121    ACCACACCGACCCCGAGAGTGAAAAAAAGACAAGGAGGAGCGACGGATCAGTCGACCGCAGAGTCTTGGTGGAGGCCGCCCACGCAGTCATTCGTCGACACCCCGTACTCGGCCACTCAC
41       T  T  P  T  P  R  V  K  K  R  Q  G  G  A  T  D  Q  S  T  A  E  S  W  W  R  P  P  T  Q  S  F  V  D  T  P  Y  S  A  T  H
            250        260        270        280        290        300        310        320        330        340        350        360
241    GGCCTCGTCCAGACCCACCCTGCAGGCTTCGGCTCCACCGGTAACAGCATAGGTGCTGGTGATCAGTTGGCCGCTGTGTCAGAGGATGGCTCGACCCTGGGCTACACGTCTCTGGACTTC
81       G  L  V  Q  T  H  P  A  G  F  G  S  T  G  N  S  I  G  A  G  D  Q  L  A  A  V  S  E  D  G  S  T  L  G  Y  T  S  L  D  F
            370        380        390        400        410        420        430        440        450        460        470        480
361    GACAAACACGGACTCGACGAGCTGAGGCGCAACCCGCTTCTGTCTTCACCCGAGGAGTGCGCCCGTGCCTGCAGAGAGGGCGAACCCCCCAGGATTTGTTACTACCACTTCACCCTCGAA
121      D  K  H  G  L  D  E  L  R  R  N  P  L  L  S  S  P  E  E  C  A  R  A  C  R  E  G  E  P  P  R  I  C  Y  Y  H  F  T  L  E
            490        500        510        520        530        540        550        560        570        580        590        600
481    CTTTACACTGTTCTCGGAGCTGCTTGCCAAGTGTGCGTTCCCAATGCGACAAACACAGTATGGAGCCACTGTCAGTGTGTCTTAGCTGATGGAGTAGAAAGAGGAATTTTGACAGTCAAC
161      L  Y  T  V  L  G  A  A  C  Q  V  C  V  P  N  A  T  N  T  V  W  S  H  C  Q  C  V  L  A  D  G  V  E  R  G  I  L  T  V  N
            610        620        630        640        650        660        670        680        690        700        710        720
601    AGAATGATCCCTGGACCTAGTATACAAGTTTGTGAAGGAGACAAAGTTGTTGTAGACGTCAGGAACCAGATGGAAGGTCTAGAAGTCACGATCCACTGGCACGGTATCACCCAACGTGGA
201      R  M  I  P  G  P  S  I  Q  V  C  E  G  D  K  V  V  V  D  V  R  N  Q  M  E  G  L  E  V  T  I  H  W  H  G  I  T  Q  R  G
            730        740        750        760        770        780        790        800        810        820        830        840
721    ACTCAGTACTCCGATGGTGTTCCCATGGTTACGCAGTGCCCCATCTTACAAGGAAACACTTTCCGGTATCAATGGTACGCGAACGCTGGTACTCACTTCTGGCACGCTCACACTGGAGTC
241      T  Q  Y  S  D  G  V  P  M  V  T  Q  C  P  I  L  Q  G  N  T  F  R  Y  Q  W  Y  A  N  A  G  T  H  F  W  H  A  H  T  G  V
            850        860        870        880        890        900        910        920        930        940        950        960
841    CAAAAGATGGATGGTATCTACGGAAGTATCGCCGTGCGACAAGCTCCTTCAGAGGACCCCAACAGCCACCTTTACGACTACGATTTAACGACTCACGTCATGCTCCTCTCGGATTGGATG
281      Q  K  M  D  G  I  Y  G  S  I  A  V  R  Q  A  P  S  E  D  P  N  S  H  L  Y  D  Y  D  L  T  T  H  V  M  L  L  S  D  W  M
            970        980        990       1000       1010       1020       1030       1040       1050       1060       1070       1080
961    CACGAAGACGCTTTGGAGAGGTTCCCCGGAAGACTTGCCGCCAACACTGGTCAGGATCCTGAGACTCTGCTCATCAACGGAAAAGGACAGTTCACGGATCCAATGACCGGCTACAGCACC
321      H  E  D  A  L  E  R  F  P  G  R  L  A  A  N  T  G  Q  D  P  E  T  L  L  I  N  G  K  G  Q  F  T  D  P  M  T  G  Y  S  T
           1090       1100       1110       1120       1130       1140       1150       1160       1170       1180       1190       1200
1081   AACACCCCCTTGGAAACATTCACGATCACTCCTGGCAGAAGGTACAGATTCCGTATGATCAACTCTTTGGCTAGTGTCTGCCCAGCTCAACTGACTGTTCAAGGTCATCCCCTCATCCTC
361      N  T  P  L  E  T  F  T  I  T  P  G  R  R  Y  R  F  R  M  I  N  S  L  A  S  V  C  P  A  Q  L  T  V  Q  G  H  P  L  I  L
           1210       1220       1230       1240       1250       1260       1270       1280       1290       1300       1310       1320
1201   ATCGCCACCGATGGAGAGCCAGTCCACCCTGTTGTTGTAAACACCATCATCTCTTTCTCAGGAGAAAGATACGATTTCGTGATCAATGCCGAACAGCCCGTCGGAGCTTACTGGATCCAA
401      I  A  T  D  G  E  P  V  H  P  V  V  V  N  T  I  I  S  F  S  G  E  R  Y  D  F  V  I  N  A  E  Q  P  V  G  A  Y  W  I  Q
           1330       1340       1350       1360       1370       1380       1390       1400       1410       1420       1430       1440
1321   GTTCGAGGACTCGGAGAATGTGGTAACAAACGAGTGCAGCAATTGGCCATCCTCCGGTACGCTCGAGGACCCTACCAACCCACTTCCCGTGCTCCCACCTACGATATTGGAATCCCACAA
441      V  R  G  L  G  E  C  G  N  K  R  V  Q  Q  L  A  I  L  R  Y  A  R  G  P  Y  Q  P  T  S  R  A  P  T  Y  D  I  G  I  P  Q
           1450       1460       1470       1480       1490       1500       1510       1520       1530       1540       1550       1560
1441   GGAGTCGTTCTTAACCCGTTGGATGCCGTCTGCAACAGACCGAGAACCGATGCCATTTGCATAAACCAATTGAAAAACGCCAAAAAGGTAGATAAGGCTTTACTCCAAGAACTGCCCGAC
481      G  V  V  L  N  P  L  D  A  V  C  N  R  P  R  T  D  A  I  C  I  N  Q  L  K  N  A  K  K  V  D  K  A  L  L  Q  E  L  P  D
           1570       1580       1590       1600       1610       1620       1630       1640       1650       1660       1670       1680
1561   ATCAAGATCTTCTTGCCGTTCAAATTCTTGTTCTACCGACCTGAGGAAATCTTCACACCACACACTTACAACAGATTCTTGGTTGCTCCGGGTGGTGGAGATCATGTCATCAGTTTAGTG
521      I  K  I  F  L  P  F  K  F  L  F  Y  R  P  E  E  I  F  T  P  H  T  Y  N  R  F  L  V  A  P  G  G  G  D  H  V  I  S  L  V
           1690       1700       1710       1720       1730       1740       1750       1760       1770       1780       1790       1800
1681   GACAACATTTCGTACGTTTCGCCACCAGCTCCACCTCTAAGTCAGATCGACGACATCCCACCAGAGATGTTCTGCAACGGAGACAACAGGCCAGCAGACTGTGGAAGGAACTGTATGTGC
561      D  N  I  S  Y  V  S  P  P  A  P  P  L  S  Q  I  D  D  I  P  P  E  M  F  C  N  G  D  N  R  P  A  D  C  G  R  N  C  M  C
           1810       1820       1830       1840       1850       1860       1870       1880       1890       1900       1910       1920
1801   ACACACAAGGTGGACATTCCTCTTCACGCTATTGTAGAGGTCGTTCTCGTCGACGAGGTACAACAACCAAACTTGAGTCATCCGTTCCATTTGCACGGTTACACATTCAATGTCATCGGT
601      T  H  K  V  D  I  P  L  H  A  I  V  E  V  V  L  V  D  E  V  Q  Q  P  N  L  S  H  P  F  H  L  H  G  Y  T  F  N  V  I  G
           1930       1940       1950       1960       1970       1980       1990       2000       2010       2020       2030       2040
1921   ATGGGTCGATCACCAGACAAGAACGTTAAGAAAATCAACTTGAAACACGCATTAGATCTTGACAGGCGAGGACTGTTGGACAGACACTTCAACCTTCCTCCCGGCAAGGACACAATTGCA
641      M  G  R  S  P  D  K  N  V  K  K  I  N  L  K  H  A  L  D  L  D  R  R  G  L  L  D  R  H  F  N  L  P  P  G  K  D  T  I  A
           2050       2060       2070       2080       2090       2100       2110       2120       2130       2140       2150       2160
2041   GTACCAAACAACGGATACGTTGTCTTTAGGTTTAGGGCAGACAATCCAGGTTACTGGTTGTTCCACTGTCACTTCTTGTTCCACATTCTCATCGGTATGAACTTAGTCCTTCACGTAGGT
681      V  P  N  N  G  Y  V  V  F  R  F  R  A  D  N  P  G  Y  W  L  F  H  C  H  F  L  F  H  I  L  I  G  M  N  L  V  L  H  V  G
           2170       2180       2190       2200       2210       2220       2230
2161   ACTCACGCCGACCTACCACCAGTGCCAGAAGGTTTCCCTCGATGTGGAGACTTCTTACCCCCGGTCAGTTTACATTGA
721      T  H  A  D  L  P  P  V  P  E  G  F  P  R  C  G  D  F  L  P  P  V  S  L  H  *
```

Figure S1. The protein-coding DNA sequence and amino acid sequence for *B. tabaci* MEAM1 *Lac2* gene. Note: start codon (ATG); stop codon (TGA); glycosylation sites are double-underlined; blue areas are signal peptide site; yellow areas represent copper ion domains

GATCGACGACATCCCACCAGAGATGTTCTGCAACGGAGACAACAGGCCAGCAGACTGTGGAAGGA
ACTGTATGTGCACACACAAGGTGGACATTCCTCTTCACGCTATTGTAGAGGTCGTTCTCGTCGACGA
GGTACAACAACCAAACTTGAGTCATCCGTTCCATTTGCACGGTTACACATTCAATGTCATCGGTATG
GGTCGATCACCAGACAAGAACGTTAAGAAAATCAACTTGAAACACGCATTAGATCTTGACAGGCG
AGGACTGTTGGACAGACACTTCAACCTTCCTCCCGGCAAGGACACAATTGCAGTACCAAACAACGG
ATACGTTGTCTTTAGGTTTAGGGCAGACAATCCAGGTTACTGGTTGTTCCACTGTCACTTCTTGTTCC
ACATTCTCATCGGTATGAACTTAGTCCTTCACGTAGGTACTCACGCCGACCTACCACCAGTGCCAGA
AGGTTTCCCTCGAT

Figure S2. The sequence of the gene region used for dsRNA syhthesis.
